# miR-301b~miR-130b—PPARγ axis underlies the adipogenic capacity of mesenchymal stem cells with different tissue origins

**DOI:** 10.1038/s41598-017-01294-2

**Published:** 2017-04-25

**Authors:** Lulu Liu, Haihui Liu, Mingtai Chen, Saisai Ren, Panpan Cheng, Hao Zhang

**Affiliations:** 1 Central Laboratory, Affiliated Hospital of Jining Medical University, Jining, 272029 Shandong Province China; 2 Department of Graduate School, Jining Medical University, Jining, 272000 Shandong Province China; 3 Department of Hematology, Affiliated Hospital of Jining Medical University, Jining, 272029 Shandong Province China

## Abstract

Mesenchymal stem cells (MSCs) have been widely used in regenerative medicine and cellular therapy due to their multi-lineage differentiation potential and immunomodulatory function. The applicability of MSCs also depends on their cellular sources and *in vivo* functions. Here in this study, we systematically compared the morphologic characteristics, immunophenotypes and the adipogenic differentiation of MSCs derived from umbilical cord (UC), adipose tissue (Ad) and bone marrow (BM). We found that the three tissues-derived MSCs displayed decreased adipogenic capacity in the order: Ad-MSC > BM-MSC > UC-MSC, and no morphologic and immunophenotypic differences were observed. Mechanistic investigation revealed a miR-301b~miR-130b—PPARγ axis, whose expression pattern in UC-MSC, Ad-MSC and BM-MSC significantly correlates with their adipogenic capacity. Our results come up with a potential mechanism to elucidate the differential adipogenesis of Ad-MSC, BM-MSC and UC-MSC, which would provide instructional advice for which source of MSCs to choose according to a certain clinical purpose. Furthermore, the miR-301b~miR-130b—PPARγ axis may also be used as a potential therapeutic target for the disorders associated with MSCs-mediated abnormal adipogenesis.

## Introduction

Mesenchymal stem cells (MSC) are a kind of plastic-adherent and fibroblast-like multipotent progenitor cells with self-renewal capacity and multi-lineage differentiation potential^1, 2^. MSCs can be induced to differentiate into osteocytes, chondrocytes and adipocytes under certain conditions and also possess immunomodulatory function with low immunogenicity, which makes MSCs a promising choice in regenerative medicine and cellular therapy^3, 4^.

MSCs were first isolated from mouse bone marrow (BM) and described by Friedenstein and colleagues in 1970s^5^, and it was until 1999 that MSCs were identified in human BM^6, 7^. However, the clinical applicability of BM-MSCs is limited due to the relatively invasive procedure required for sample collection as well as the low frequency of MSCs in the BM mononuclear cells^8, 9^. Therefore, investigators turned to substitute tissues of BM as sources of MSCs, such as dental pulp, placenta, umbilical cord and adipose tissue^10, 11^. Current research on MSCs is mainly focused on their self-renewal capacity, multi-lineage differentiation potential, surface markers, and immune regulation^12–14^. Multiple comparative studies have demonstrated that MSCs derived from different tissues have varied differentiation potential, proliferative ability and immunomodulatory effect, in spite of their similar morphological characteristics and surface antigen expression^15–17^. However, the detailed mechanism underlying the differences remains to be determined.

Here in this study, we isolated MSCs from umbilical cord (UC-MSC), adipose tissue (Ad-MSC) and bone marrow (BM-MSC), compared their adipogenic ability and explored the intrinsic mechanism underlying the differences in term of the gene expression. We found that the adipogenic capacity decreased in the order Ad-MSC > BM-MSC > UC-MSC. Peroxisome proliferative activated receptor gamma (PPARγ), an essential transcriptional factor of adipogenesis^18^, showed decreased expression in the order: Ad-MSC > BM-MSC > UC-MSC, which positively correlates with the adipogenic capacity of the three tissues-derived MSCs. MiR-301b~miR-130b cluster, among of which miR-130b has been reported to inhibit adipogenesis by repressing PPARγ expression, presented a expression pattern negatively correlating with PPARγ expression in the three tissues-derived MSCs. Thus, our results established a miR-301b~miR-130b—PPARγ axis whose expression pattern, to some degree, elucidated the differential adipogenic capacity of the three tissues-derived MSCs.

## Results

### Morphological observations of the MSCs derived from different tissues

**Figure 1:**
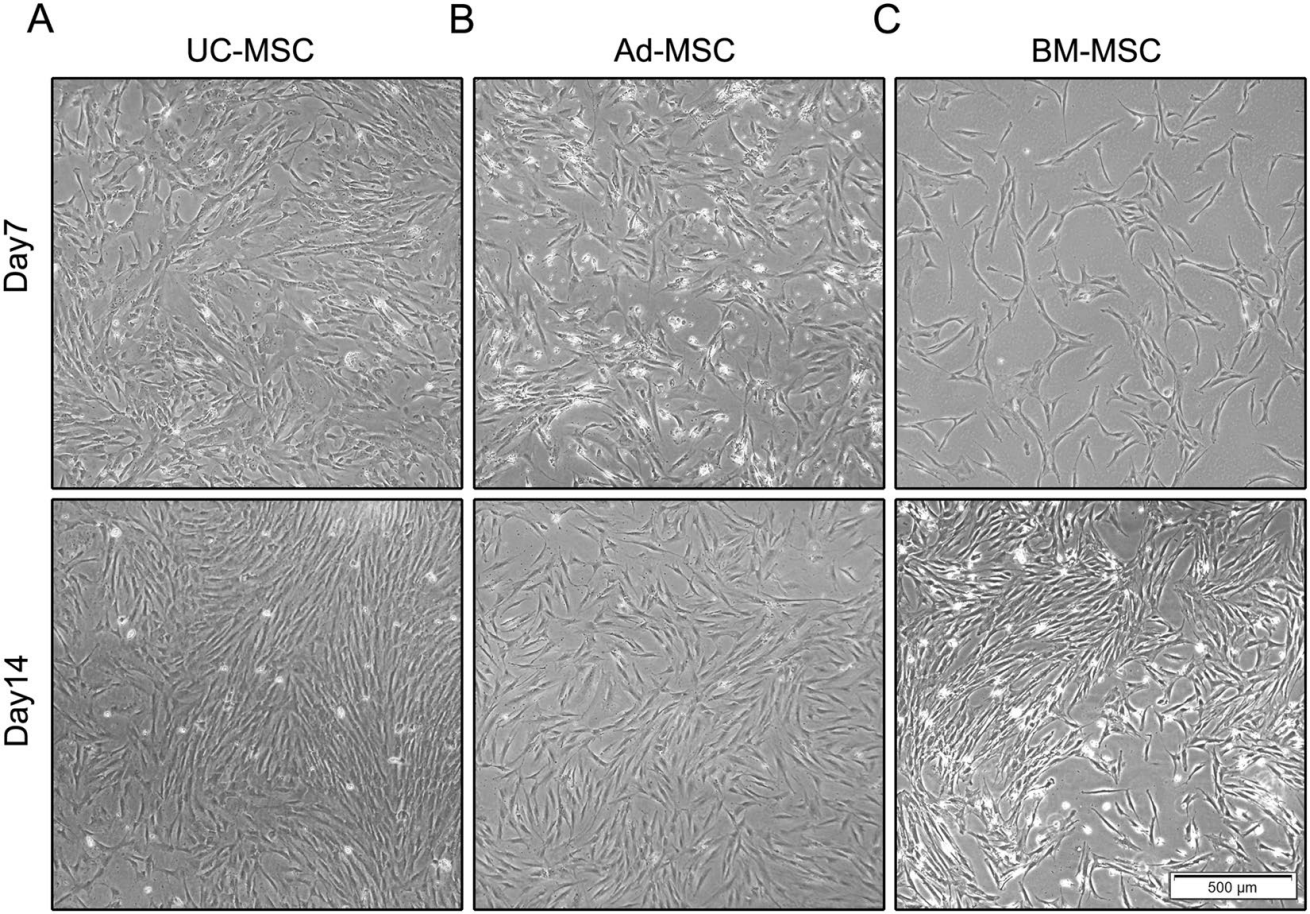
Morphologic comparison of UC-MSC, Ad-MSC and BM-MSC. UC-MSC. (**A**) Ad-MSC (**B**) and BM-MSC (**C**) were isolated and captured using the IX71 Olympus microscope at day 7 and day 14.

We successfully isolated MSCs from umbilical cord, adipose tissue and bone marrow from the healthy donors. The morphology of UC-MSC, Ad-MSC and BM-MSC were visualized under phase contrast microscope (Fig. 1). The three tissues-derived MSCs were found to be similar at the indicated culture days and no obvious morphologic differences were observed. However, UC-MSC and Ad-MSC are superior to BM-MSC to get the same confluence for the first passage. Furthermore, UC-MSC and Ad-MSC presented stronger proliferative ability than BM-MSC according to our experience, which may make umbilical cord and adipose tissue ideal substitutes for bone marrow to isolate MSCs for the clinical application.

### Immunophenotype characterization of the three tissues-derived MSCs

**Figure 2:**
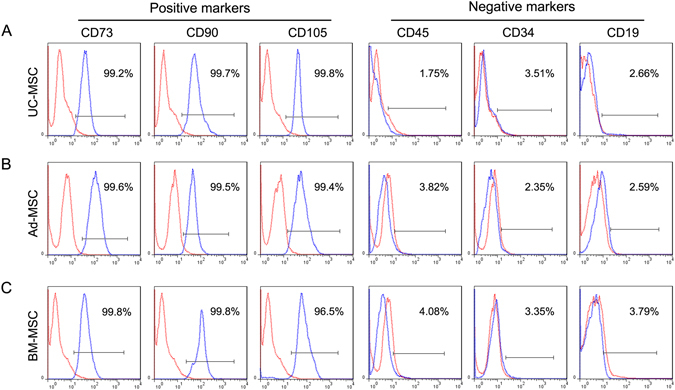
Immunophenotype of MSCs. UC-MSC (**A**), Ad-MSC (**B**) and BM-MSC (**C**) were labeled with antibodies against the indicated antigens, and analyzed by flow cytometry. The markers CD73, CD90 and CD105 showed highly positive expression and the markers CD45, CD34 and CD19 showed negative expression, consistent with the characteristic pattern of mesenchymal surface markers.

To determine whether the adherent cells from umbilical cord, adipose tissue and bone marrow met the quantifying criteria of MSCs, we analyzed the expression of surface antigen (CD73, CD90, CD105, CD45, CD34 and CD19). As shown in Fig. 2, stromal cell markers (CD73, CD90 and CD105) were expressed in UC-MSC, Ad-MSC and BM-MSC, with a high positivity rate. However, hematopoietic cell markers (such as CD45, CD34 and CD19) were not expressed. All the surface markers expression detected in the three tissues-derived MSCs conformed to the criteria of MSCs established before^19^ and no comparable differences were observed among UC-MSC, Ad-MSC and BM-MSC.

### Adipogenic differentiation of UC-MSC, Ad-MSC and BM-MSC

**Figure 3:**
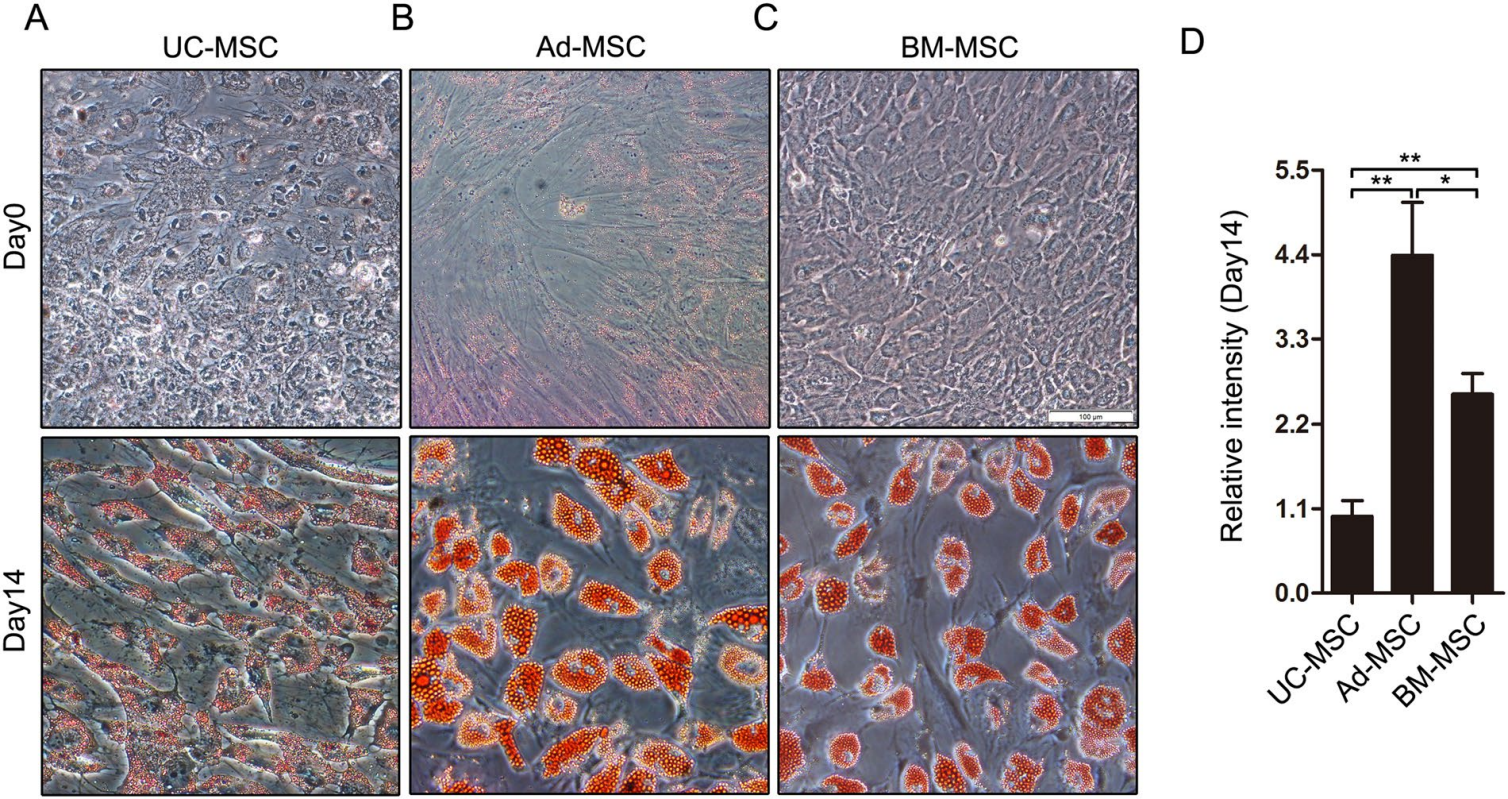
Comparison of adipogenic differentiation in the three tissues-derived MSCs by oil red O staining. UC-MSC, Ad-MSC and BM-MSC were incubated with or without adipogenic medium for 14 days. Then the cells were stained with oil red O and captured using the IX71 Olympus microscope. The staining of UC-MSC (**A**), Ad-MSC (**B**) and BM-MSC (**C**) at Day 0 and Day 14 of adipogenic differentiation was presented. (**D**) The relative quantification of the staining at Day 14 was analyzed and shown. *P < 0.05 and **P < 0.01, Student’s t-test.

**Figure 4:**
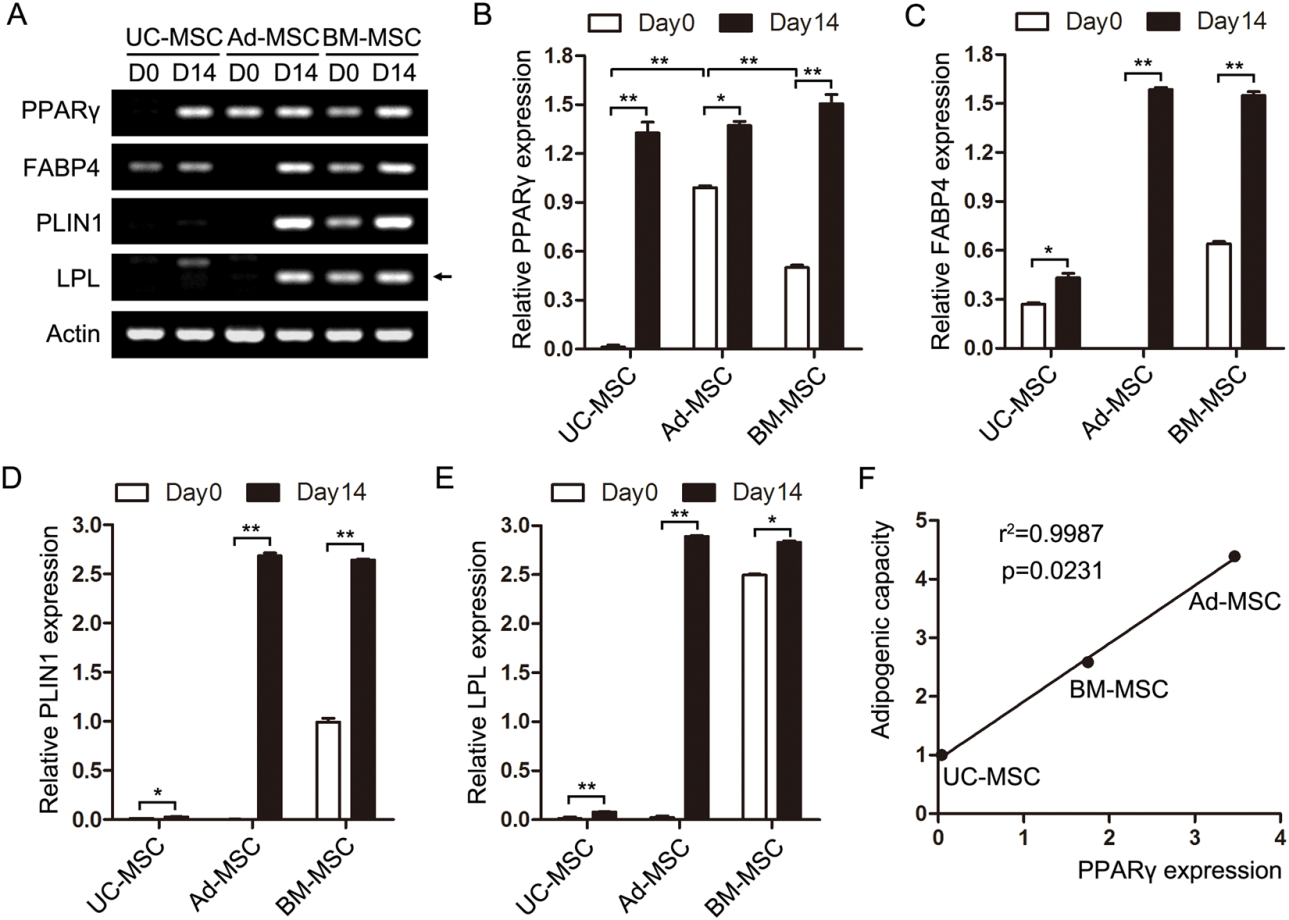
Comparison of adipogenic gene expression in the three tissues-derived MSCs by RT-PCR. UC-MSC, Ad-MSC and BM-MSC were incubated with or without adipogenic medium for 14 days. (**A**) Adipogenic markers including PPARγ, FABP4, PLIN1 and LPL were detected at the indicated days of differentiation. Actin was used as a loading control. The picture was cropped and adjusted using photoshop software and the original image was presented in Supplementary Fig. 1. (**B**–**E**) The quantification of the four marker genes was performed using Gel-Pro software and presented. (**F**) The correlation of adipogenic capacity with PPARγ expression in the three sources-derived MSCs was performed using linear regression analysis. *P < 0.05 and **P < 0.01, Student’s t-test.

Adipogenic differentiation under certain condition is a basic feature of MSCs. Multiple comparative studies have been performed about the MSCs’ multi-lineage differentiation potential including adipogenic differentiation^15^. However, the adipogenic capacity of UC-MSC, Ad-MSC and BM-MSC is still controversial. Here, passage 3 or passage 4 of UC-MSC, Ad-MSC and BM-MSC were chosen and seeded into 12-well plate followed by adipogenic induction for 14 days when the cells reached 90% confluence. Then the cells were fixed and stained with oil red O. As presented in Fig. 3A–C, UC-MSC, Ad-MSC and BM-MSC all showed increased lipid vesicles at differentiation day 14 compared with their respective controls (differentiation day 0). At differentiation day 0, no obvious differences of oil red O staining among the three tissues-derived MSCs were observed. However, at differentiation day 14, the intensity of oil red O staining can be arranged from strong to weak: Ad-MSC > BM-MSC > UC-MSC (Fig. 3D). Next, the adipogenic differentiation markers (PPARγ, FABP4, PLIN1 and LPL) were detected using semi-quantitative RT-PCR at differentiation day 0 and day 14 (Fig. 4A). And the gray density of the four genes expression were also analyzed and presented in Fig. 4B–D, which, to some degree, further confirmed the adipogenic capacity of the three tissues-derived MSCs (Ad-MSC > BM-MSC > UC-MSC). Both Ad-MSC and BM-MSC have strong adipogenic differentiation potential, with Ad-MSC slightly stronger than BM-MSC according to the oil red O staining result. Both their adipogenic capacity are much stronger than that of UC-MSC based on the staining and PCR results.

### MiR-301b~miR-130b—PPARγ expression pattern correlates with adipogenic capacity of UC-MSC, Ad-MSC and BM-MSC

**Figure 5:**
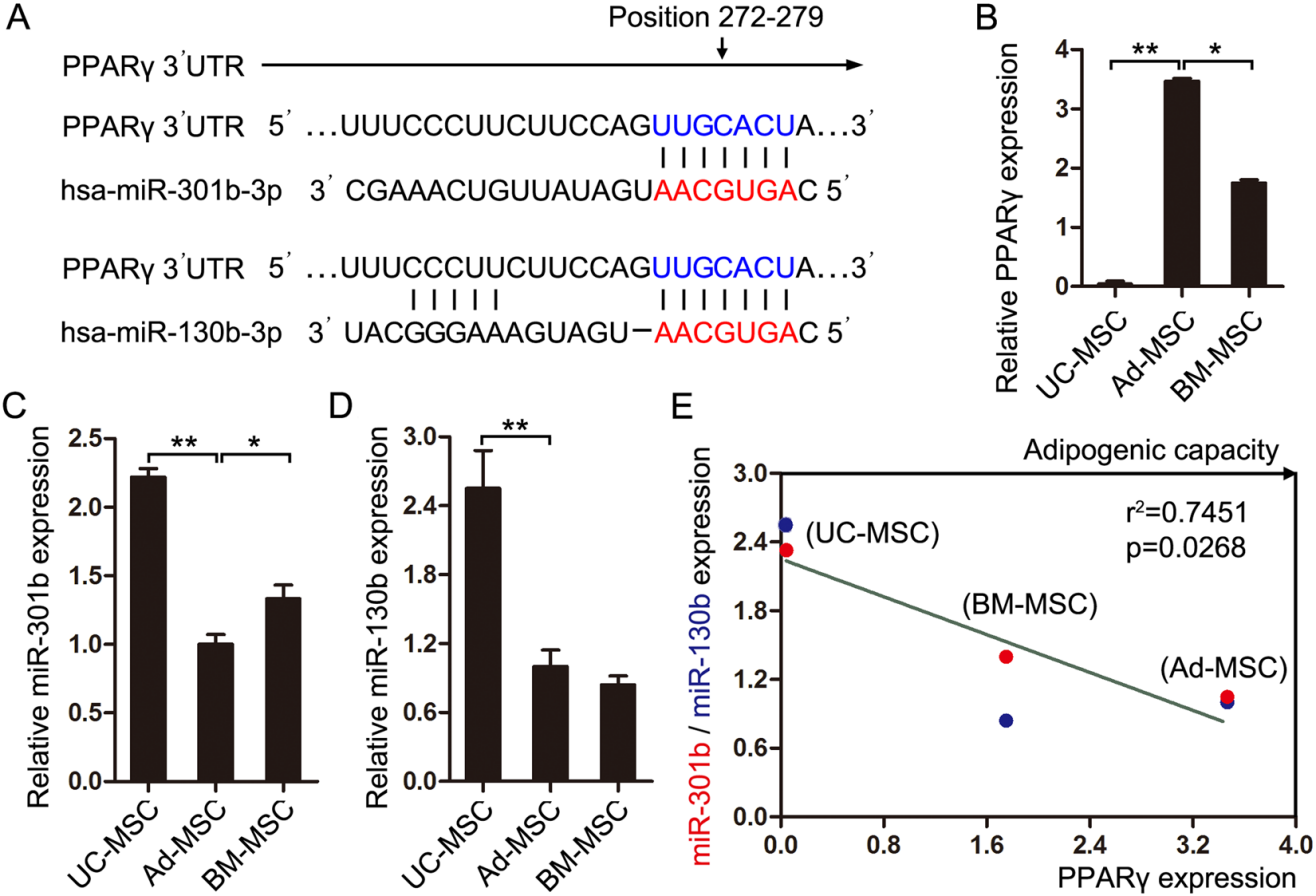
MiR-301b~miR-130b—PPARγ expression pattern correlate with the adipogenic capacity of the three sources-derived MSCs. (**A**) The binding sites of miR-301b~miR-130b in PPARγ 3′UTR were shown. (**B**–**D**) The expression of PPARγ, miR-301b and miR-130b were detected in UC-MSC, Ad-MSC and BM-MSC. (**E**) The correlation of miR-301b/miR-130b expression with PPARγ expression in the three sources-derived MSCs was performed using linear regression analysis. *P < 0.05 and **P < 0.01, Student’s t-test.

To reveal the potential mechanism underlying the differences of adipogenic capacity of UC-MSC, Ad-MSC and BM-MSC, we unexpectedly found that the original expression of PPARγ (Fig. 4A,B), which is an essential transcription factor for adipogenesis^20^, in the three tissues-derived MSCs, positively correlates with their adipogenic potential (Fig. 4F). Next we want to know what determines the differential expression of PPARγ in UC-MSC, Ad-MSC and BM-MSC. We first turned to microRNAs which are a class of 21–23 nt small non-coding RNAs and can post-transcriptionally regulate gene expression by binding to the 3′-untranslated region (3′UTR) of target mRNAs through their seed sequences^21^. MiRNAs have also been intensively studied in the past few years and can participate in the regulation of many physiological processes including adipogenesis^22^. Thus, using TargetScan and PicTar web-tools, we predicted the miRNAs that may target PPARγ expression. Combined with miRNAs that have been reported to target PPARγ during adipogenesis, we chose some for subsequent experimental validation and found that miR-301b~miR-130b cluster may act as a potential regulator of PPARγ expression and further the adipogenesis of UC-MSC, Ad-MSC and BM-MSC. The binding of miR-301b~miR-130b cluster to the 3′UTR of PPARγ was presented in Fig. 5A. And the expression of PPARγ and miR-301b~miR-130b cluster in UC-MSC, Ad-MSC and BM-MSC were detected by real-time quantitative PCR and presented in Fig. 5B–D. PPARγ expression was in agreement with the semi-quantitative PCR result (Fig. 5B). The expression of miR-301b increased in the order: Ad-MSC < BM-MSC < UC-MSC (Fig. 5C), which is contrary to that of PPARγ. MiR-130b presented high expression in UC-MSC, but displayed similar expression in Ad-MSC and BM-MSC (Fig. 5D). Linear regression analysis showed that miR-301b~miR-130b cluster expression presented significant negative correlation with PPARγ expression and the adipogenic capacity of UC-MSC, Ad-MSC and BM-MSC (Fig. 5E), which, to some degree, revealed a possible mechanism to elucidate the varied adipogenic potentials of the three tissues-derived MSCs.

## Discussion

MSCs could be isolated from many adult tissues and fetal appendages without too much ethical controversy and have been widely used to repair or regenerate damaged tissues and treat immune diseases in clinical trials also due to their multi-lineage differentiation potential and immunomodulatory function^7, 23^. MSCs derived from different tissues may have varied functional properties in spite of the similar morphologic characteristics and immunophenotypes, which make it complicated and confusing about what a source of MSCs to choose for a certain clinical purpose^15, 17^. Many comparative studies have been performed to investigate the proliferation, differentiation and immune regulation of MSCs derived from different tissues. For example, Ad-MSCs have been shown to possess higher proliferative and angiogenic capabilities *in vitro* as compared to the BM-MSCs^15, 24^. Choong *et al*. reported that BM-MSCs were also shown to undergo senescence at an earlier population doubling than the adipose and umbilical cord tissue-derived stem cells from equine tissue^25^. Placenta-derived MSCs were shown to have a lower potential to undergo adipogenesis but have a higher potential to undergo osteogenesis than BM-MSCs and Ad-MSCs^17^. However, the comparative adipogenic potentials of UC-MSC, Ad-MSC and BM-MSC are still controversial and the underlying molecular mechanism remains to be explored. Here in this study, we systematically compared the morphologic characteristics, surface antigen expression and adipogenic differentiation of UC-MSC, Ad-MSC and BM-MSC. We found that the three tissues-derived MSCs have similar morphology and immunophenotypes (Figs 1 and 2) as reported, but displayed decreased adipogenic capacity in the order Ad-MSC > BM-MSC > UC-MSC (Figs 3 and 4).

Adipogenesis is an important physiological process to produce adipocytes which play a vital role in energy homeostasis and process the largest energy reserve as triglycerol in the body of animals^26^. MSCs mediated adipogenesis is also essential to maintain the local microenvironment of the organisms and has been intensively studied in the past few decades^27–29^. The process is tightly controlled by transcription factors and noncoding RNAs^30–32^. To reveal the underlying mechanism of differential adipogenic capacity of UC-MSC, Ad-MSC and BM-MSC, we unexpectedly found that PPARγ mRNA expression in the three tissues-derived MSCs positively correlates with their adipogenic capacity. PPARγ has been acknowledged as an essential transcription factor for adipogenic lineage commitment^20, 33^.

To further seek what determines the differential expression of PPARγ in UC-MSC, Ad-MSC and BM-MSC, we first turned to the reported miRNAs which play important roles in regulating adipogenesis. Many miRNAs have been identified in different models of adipogenic differentiation, and miR-27 and miR-130b were reported to inhibit PPARγ expression during MSCs-mediated adipogenesis^34–36^. MiR-27 and miR-130b were first chosen and their expression was detected in UC-MSC, Ad-MSC and BM-MSC. No significant negative correlation was observed between miR-27 expression and PPARγ expression in the three tissues-derived MSCs (Supplementary Fig. 2). Fortunately, miR-130b had inversed expression with that of PPARγ in UC-MSC and Ad-MSC, whereas miR-130b displayed similar expression in Ad-MSC and BM-MSC (Fig. 5D). MiR-130/miR-301/miR-454 family has been reported to be regulated together to exert a biological effect because of their same seed sequences^37^. Thus, the other family members, miR-130a, miR-301a, miR-301b and miR-454, were also chosen and their expression was detected in the three tissues-derived MSCs. Only miR-301a and miR-301b displayed a similar expression pattern as miR-130b. However, linear regression analysis showed no significant negative correlation between PPARγ expression and the respective expression of all the family members including miR-130b (Supplementary Fig. 3). Both miR-301b and miR-130b are located in chromosome 22 and belong to the miR-301b~miR-130b cluster^38, 39^. When they were analyzed as a cluster, their expression displayed a significant negative correlation with PPARγ expression (Fig. 5E). We first uncovered a miRNA cluster whose expression underlies PPARγ expression and adipogenic potential of the three tissues-derived MSCs.

Our study demonstrated that MSCs derived from umbilical cord, adipose tissue and bone marrow displayed decreased adipogenic capacity in the order Ad-MSC > BM-MSC > UC-MSC. We also established a miR-301b~miR-130b—PPARγ axis whose expression pattern has a significant correlation with the adipogenic potential of the three tissues-derived MSCs, which may provide a potential therapeutic target for the disorders associated with MSCs-mediated abnormal adipogenesis, and provide instructional advice for which source of MSCs to choose according to a certain clinical purpose.

## Methods

### Isolation and culture of human MSCs

Human umbilical cord, adipose tissue and bone marrow from healthy donors were collected from the affiliated hospital of Jining Medical University. All human MSC studies were approved by the Ethics Committees of the hospitals and the Institutional Review Board of Jining Medical University and carried out in accordance with their approved guidelines and all participants provided written informed consents. MSCs were isolated from the three tissues as previously described^40–42^. All the cells were cultured in Dulbecco’s modified Eagle’s medium (DMEM) supplemented with 10% fetal bovine serum (FBS) (Gibco).

### Flow cytometry analysis

UC-MSC, Ad-MSC and BM-MSC were harvested at passage 3. The cells were rinsed twice with PBS and re-suspended in 100 µl PBS. Then the cells were incubated with mouse anti-human CD73/CD90/CD105/CD45/CD34/CD19 (Biolegend) at 4 °C for 30 min. The cells were washed with 1 ml PBS, re-suspended in 300 µl PBS and analyzed by flow cytometry (BD Biosciences, USA). The mouse IgG1-isotype was used as a negative control.

### Adipogenic differentiation

Adipogenic differentiation was performed according to the method described elsewhere with minor modifications^40^. The MSCs were seeded into 12/6-well plates. Upon reaching confluence, the MSCs were changed into adipogenic medium composed of DMEM with 10% FBS, 1 µM dexamethasone, 0.01 mg/mL insulin, 100 µg/mL indomethacin and 0.5 mM 3-isobutyl-1-methyl-xanthine for 14 days (all purchased from Sigma, St. Louis, Mo, USA).

### Oil red O staining

The cells were rinsed with PBS twice in the plates after discarding the supernatant and fixed with 4% paraformaldehyde at room temperature for 20 min. The cells were then washed with PBS and stained with oil red O (Solarbio) for 20 min followed by washing with PBS. Lipid vesicles were observed and photographed using an IX71 Olympus microscope (Olympus, Tokyo, Japan). Quantification of the staining was performed using Image-Pro Plus software.

### RNA extraction and RT-PCR analysis

Total RNA was extracted from cell samples using TRIzol Reagent (Invitrogen) and quantified using the NanoDrop 2000 spectrophotometer (Thermo Scientific, Bremen, Germany). The first strand of cDNA was synthesized using M-MLV reverse transcriptase (Invitrogen) according to the manufacturer’s instruction. Oligo (dT) was used as the primers for reverse transcription of mRNA. Stem-loop RT primers were used for the reverse transcription of miRNAs. Actin and U6 were used as their respective controls. Semi-quantitative PCR and real-time quantitative PCR were performed in ABI PCR machines using pre-stained mix (Tiangen) and SYBR Premix (TransGen Biotech) respectively. The primers used for reverse transcription and RT-PCR were listed in Table 1.

**Table 1:** Primers used for reverse transcription and RT-PCR.

Name	Sequences
FABP4-F	ACTGGGCCAGGAATTTGACG
FABP4-R	CTCGTGGAAGTGACGCCTT
PPARγ-F	GCTGACCAAAGCAAAGGCG
PPARγ-R	GCCCTGAAAGATGCGGATG
LPL-F	TCATTCCCGGAGTAGCAGAGT
LPL-R	GGCCACAAGTTTTGGCACC
PLIN1-F	GCGAGGATGGCAGTCAACAAA
PLIN1-R	GCACGCCCTTCTCATAGGCAT
Actin-F	CATGTACGTTGCTATCCAGGC
Actin-R	CTCCTTAATGTCACGCACGAT
miR-301b-RT	GTCGTATCCAGTGCAGGGTCCGAGGTATTCGCACTGGATACGACGCTTTGA
miR-130b-RT	GTCGTATCCAGTGCAGGGTCCGAGGTATTCGCACTGGATACGACATGCCCT
miR-301b-Up	GCGGCGCAGTGCAATGATATT
miR-130b-Up	GTCGTGCAGTGCAATGATGAAA
miRNA-Dn	TCCAGTGCAGGGTCCGAGGT
U6-RT	AAAATATGGAACGCTTCACGAA
U6-Up	CTCGCTTCGGCAGCACATATA
U6-Dn	ACGCTTCACGAATTTGCGTGTC

### Statistical Analysis

Student’s t-test (two-tailed) was performed to analyze the data. Statistical significance was set at *P* < 0.05, as indicated by an asterisk (*P < 0.05; **P < 0.01).

## Electronic supplementary material

Supplementary Information
