# Supplementary material for: miR-301b~miR-130b—PPARγ axis underlies the adipogenic capacity of mesenchymal stem cells with different tissue origins
Source: Sci Rep. 2017 Apr 25;7:1160. doi: 10.1038/s41598-017-01294-2 (PMC5430834; doi:10.1038/s41598-017-01294-2)
Supplement: Supplementary file 1 — Supplementary Information [file 41598_2017_1294_MOESM1_ESM.pdf]

# Supplementary Information

**miR-301b~miR-130b—PPARγ axis underlies the adipogenic capacity of mesenchymal stem cells with different tissue origins**

Lulu Liu[1,4], Haihui Liu[2,4], Mingtai Chen[1,4], Saisai Ren[2], Panpan Cheng[3], Hao Zhang[3*]

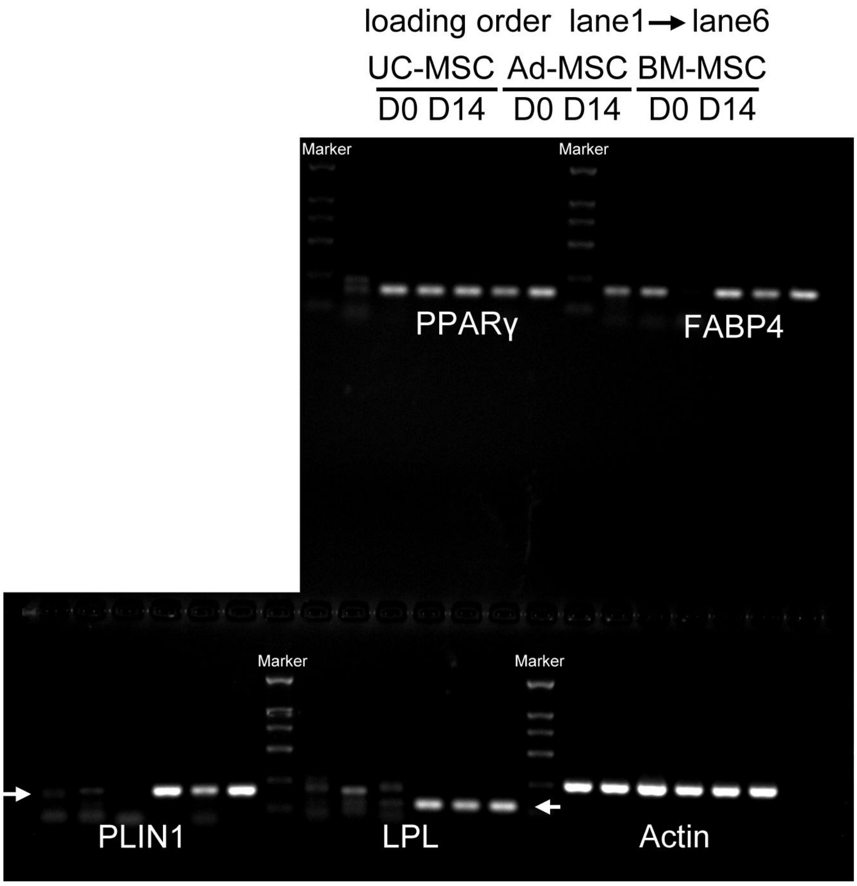


Supplementary Figure 1. Comparison of adipogenic gene expression in the three tissues-derived MSCs by RT-PCR. UC-MSC, Ad-MSC and BM-MSC were incubated with or without adipogenic medium for 14 days. Adipogenic markers including PPARγ, FABP4, PLIN1 and LPL were detected at the indicated days of differentiation. Actin was used as a loading control. (Original picture)

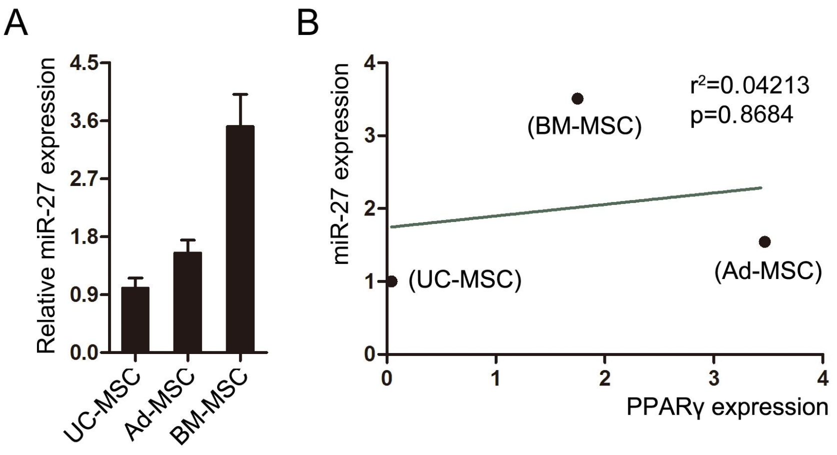


Supplementary Figure 2. Correlation analysis of miR-27 expression with PPARγ expression in the three sources-derived MSCs. (A) The expression of miR-27 was detected in UC-MSC, Ad-MSC and BM-MSC. (B) The correlation of miR-27 expression with PPARγ expression in the three sources-derived MSCs was performed using linear regression analysis.

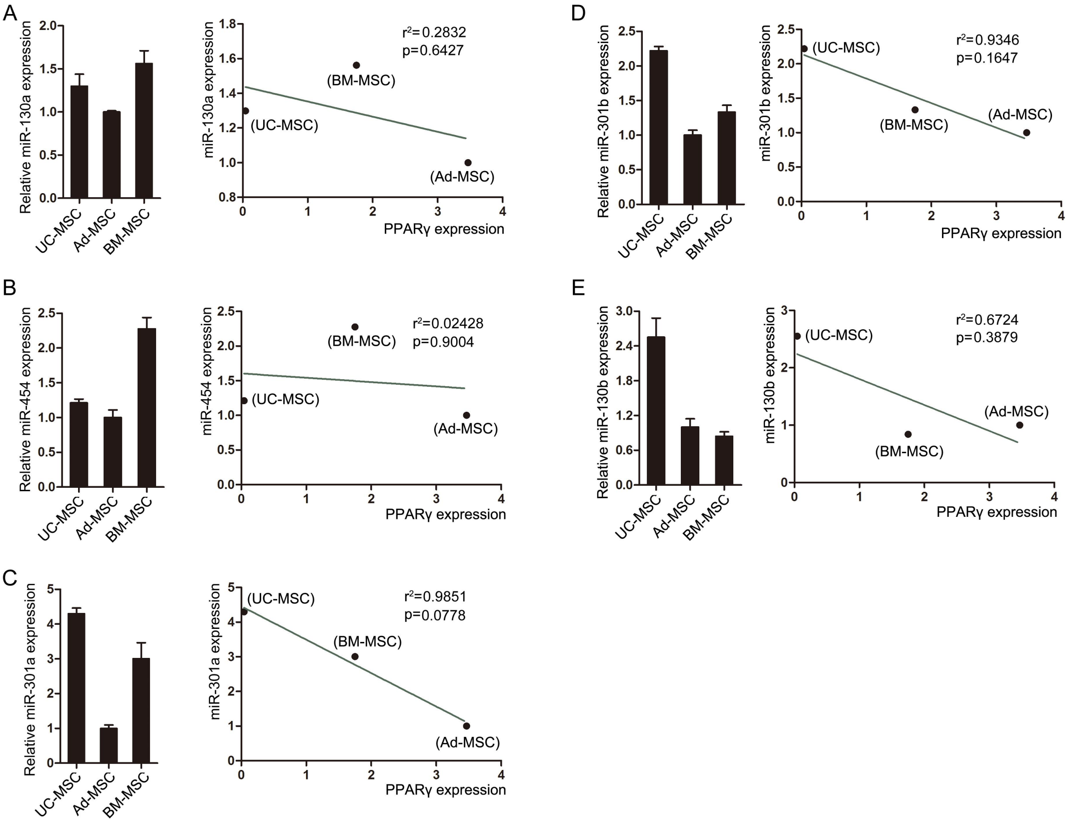


Supplementary Figure 3. Correlation analysis of miR-130/301/454 family expression with PPARγ expression in the three sources-derived MSCs. (A) The expression of miR-130a and its correlation with PPARγ expression in UC-MSC, Ad-MSC and BM-MSC. (B) The expression of miR-454 and its correlation with PPARγ expression in UC-MSC, Ad-MSC and BM-MSC. (C) The expression of miR-301a and its correlation with PPARγ expression in UC-MSC, Ad-MSC and BM-MSC. (D) The expression of miR-301b and its correlation with PPARγ expression in UC-MSC, Ad-MSC and BM-MSC. (E) The expression of miR-130b and its correlation with PPARγ expression in UC-MSC, Ad-MSC and BM-MSC.
